# Exploring the Distinct Distribution of Archaeal Communities in Sites Contaminated with Explosives

**DOI:** 10.3390/biom12040489

**Published:** 2022-03-23

**Authors:** Yash Pal, Shanmugam Mayilraj, Srinivasan Krishnamurthi

**Affiliations:** Microbial Type Culture Collection & Gene Bank (MTCC), CSIR-Institute of Microbial Technology, Sec-39A, Chandigarh 160036, India; pal.yashlap@gmail.com

**Keywords:** amplicon sequencing, *Archaea*, MEGAN, RDX (1,3,5-Trinitro-1,3,5-triazine), HMX (1,3, 5,7-Tetranitro-1,3,5,7-tetrazocane)

## Abstract

Most of the research on bioremediation and estimation of microbial diversity in waste contaminated sites is focused on the domain *Bacteria*, whereas details on the relevance of *Archaea* are still lacking. The present study examined the archaeal diversity and predicted metabolic pathways in two discrete sites (SITE1 and SITE2) contaminated with explosives (RDX and HMX) by amplicon-targeted sequencing of 16S rRNA genes. In total, 14 soil samples were processed, and 35,758 OTUs were observed, among which 981 OTUs were classified as *Archaea*, representing ~2.7% of the total microbial diversity in our samples. The majority of OTUs belonged to phyla *Euryarchaeota* (~49%), *Crenarchaeota* (~24%), and *Thaumarchaeota* (~23%), while the remaining (~4%) OTUs were affiliated to *Candidatus* Parvarchaeota, *Candidatus* Aenigmarchaeota, and *Candidatus* Diapherotrites. The comparative studies between explosives contaminated and agricultural soil samples (with no history of explosives contamination) displayed significant differences between the compositions of the archaeal communities. Further, the metabolic pathways pertaining to xenobiotic degradation were presumably more abundant in the contaminated sites. Our data provide a first comprehensive report of archaeal communities in explosives contaminated sites and their putative degradation role in such ecosystems which have been as yet unexplored.

## 1. Introduction

*Archaea* accounts for ~1–5% of all prokaryotes in soil surface layers and substantially impacts the biogeochemical cycling process [1,2]. Archaeal processes are of particular interest in “extreme” environments since they have evolved a range of energy metabolisms, including carbon fixation from inorganic sources [2]. In addition to the extremophilic *Archaea*, several non-extremophilic lineages have been detected in environments varying from marine to terrestrial using culture-independent techniques such as stable isotope probing (SIP), catalyzed reporter deposition-fluorescence in situ hybridization (CARD-FISH), and amplicon targeted sequencing [3]. Studies concerning the global distribution of dominant *Archaea* have revealed *Crenarchaeota* and *Euryarchaeota* in samples collected from soil, sediments, and water [1,4,5,6,7]. Interestingly, a few members of *Euryarchaeota*, in particular, *haloarchaea* and methanogens, are known to degrade xenobiotic pollutants and crude oil in the soil and marine sediments [8,9].

Among the explosives, RDX (research developed explosive; 1,3,5-Trinitro-1,3,5-triazine) and HMX (high melting explosive; 1,3,5,7-Tetranitro-1,3,5,7-tetrazocane) are the most widely used compounds for military operations and detonation [10,11]. RDX and HMX belong to nitramines class of explosives and hexamine is the major constituent for production of both the compounds. The low octanol-water partition coefficient of HMX (0.16, log K_ow_) and RDX (0.87, log K_ow_) suggests that they are not robustly absorbed by the organic molecules present in soil/sediment and have a tendency to migrate through groundwater flow [12]. As a result of increasing demands of explosives in military operations and their careless disposal, an overwhelming amount of explosives is released in soil and water ecosystems and exerts a noxious effect on the surrounding ecosystems. The effect of high and low concentrations of environmental contaminants and variables can range from total inhibition to bio-stimulation of some groups of native microbial communities [13,14]. Previous studies on evaluation of microbial community composition at explosives contaminated sites have utilized both the culture-dependent [15,16,17,18] and culture-independent [19,20,21,22,23] methods. Although in both intrinsic and engineered bioremediation processes, Archaea are often involved along with bacteria, unfortunately most of the above research has focused on the latter domain [22,23]. In addition, the sequencing protocol, including the primer specificity, depth of sequencing, sequencing platforms, varied significantly between the studies [21,22,23,24]. Similar disparity exists among other studies practicing RFLP (restriction fragment length polymorphism), DGGE (denaturing gradient gel electrophoresis), and SIP (stable isotope probing) techniques for the estimation of microbial diversity in explosives contaminated sites [19,20,23]. Indeed, very little is known about the diversity and distribution of archaeal communities compared to bacterial diversity and function in explosives contaminated sites.

For the present study, we investigated and compared the archaeal community structure and its distribution using NGS based meta-barcoding analysis of 16S rRNA gene at two geographically distinct explosives contaminated sites. Our findings suggest that the archaeal community composition at explosives contaminated site was significantly different from the agricultural soil samples. Until the time of writing this manuscript and to our knowledge, this is the first detailed evaluation and comparison of archaeal diversity from explosives contaminated sites.

## 2. Materials and Methods

### 2.1. Sample Collection

Soil samples were collected from two explosives (RDX/HMX) contaminated sites located at Central (SITE1; samples labeled as NS) and Northern (SITE2; samples labeled as PS) parts of India in the month of January and April 2016, respectively. Moreover, SITE2 has previously been studied for the isolation of explosives degrading bacteria [25]. Soil samples were collected in whirl pack bags (1L, Hi-Media, Mumbai, India) and transported to the laboratory within 4 h of sampling and immediately stored at −20 °C until further processing. Raw sequencing reads under the accession number PRJNA635685, experiment, SRX8429566 (labled as C1) and SRX8429565 (labled as C2) [26], from Rodale institute’s farming systems trial, running since 1981, with no history of exposure to explosive compounds were used as control samples to exclude their influence on microbial community structure during comparative analysis with explosives contaminated sites.

### 2.2. Analytical Techniques

Standard protocols described by Baruah and Barthakur [27] and AOAC 990.08 [28] specifications were used to determine the physicochemical parameters of only two samples with highest concentration of explosives contamination (NS2 sample for SITE1 and PS2 sample for SITE2). The concentration of explosives in all samples was detected as per U.S, EPA, 8330 methods [29]. Briefly, the soil samples were analyzed using LC-20, HPLC system (Shimadzu, Kyoto, Japan) equipped with Restek ultra C18 column (25 cm × 4.6 mm) (Restek, Bellefonte, PA, USA) and a UV-photodiode array detector (9926). The mobile phase consisted of 46% methanol (Merck, Darmstadt, Germany) and 54% deionised water (Merck, Germany) at 1 mL/min flow rate. The sample injection volume used was 10 µL with absorption detection at 254 nm. Samples quantification was performed using EPA certified RDX/HMX standards (Cat 31450; Restek, Bellefonte, PA, USA,) and known concentrations of RDX and HMX.

### 2.3. DNA Extraction and High-Throughput Sequencing

According to the manufacturer’s instructions, total community DNA from soil samples was isolated using a PowerSoil DNA isolation kit (MO-BIO, Carlsbad, CA, USA). The purity and concentration of isolated DNA were accessed using NanoDrop spectrophotometer (Nanodrop Technologies Inc., Wilmington, DE, USA) and agarose gel electrophoresis (365 nm, UV-A). Targeted amplicon sequencing was performed using Illumina sequencing platform (Hi-Seq 2500). Briefly, about 4 ng of total community DNA was used for specifically amplifying V3-V4 region with *Archaea* specific primers (Arch-349F, Forward primer: 5′ TCGTCGGCAGCGTCAGATGTGTATAAGAGACAGGTGYCAGCM-GCCGCGGTAA 3′; Arch-519R, Reverse primer: 5′ GTCTCGTGGGCTCGGAGATGTGT-ATAAGAGACAGGGACTCANVGGGTWTCTAAT 3′) [30] containing a ‘tag’ sequence (F; TCGTCGGCAGCGTCAGATGTGTATAAGAGACAG, R; GTCTCGTGGGCTCGGAGATGTGTATAAGAGACAG) complementary to adapter and index primers from the Nextera XT Index kit V2 resulting in the generation of single amplicons of ~265 × 2 bp. The quality of amplified products was checked on the agarose gel before proceeding for the next round of PCR (indexing PCR). The Illumina sequencing adapters and dual indexing barcodes were further added, using limited cycle PCR, resulting in ~305 × 2 bp PCR product. The quality of the library was validated by running an aliquot (1:10) on High Sensitivity Bioanalyzer Chip (Agilent, Santa Clara, CA, USA), and the sequences were determined using Hiseq 250 Rapid-Run, using TruSeq dual index primers. The raw reads from SITE1 and SITE2 were submitted in the NCBI SRA database under Bioproject PRJNA391401.

### 2.4. Sequence and Statistical Analyses

The Illumina paired end reads were demultiplexed using bcl2fastq, and quality checked using FastQC2. The raw reads having primer sequence and high-quality bases were selected and checked for sequence accuracy using Phred quality score, and the high quality reads with more than 70% of bases and Q score >20 were considered for further analysis. The quality reads were further stitched using Fastq-join3 command. These stitched reads were considered for further analysis using USEARCH version 10.24 [31]. Briefly, the reads were quality filtered, trimmed, and the chimeric reads were removed before unique OTU identification and clustering. The OTU files from USEARCH analysis were further used for classification using RDPclassifier version 2.11, database release 11.5 (≥97% sequence similarity) [32]. The assigned archaeal OTUs were manually segregated from the output file for each sample. The OTU files with only archaea assigned taxonomy were further used for graphical representation using MEGAN version 6 [33]. The *Bacteria*:*Archaea* ratio was calculated manually using the classified OTUs (*Bacteria* or *Archaea*) from RDPclassifier output. Co-occurring taxa were determined in MEGAN using the Jaccard correlation coefficient with an edge threshold of 70%, while the core-biome representatives were determined using a sample threshold of 50%. Calculation of diversity indexes, i.e., Shannon, Simpson, Chao1, observed species, and rarefaction curves were performed using MEGAN and PAST software version 4.03 [34]. Similarity or dissimilarity between archaeal taxa of contaminated and agricultural soil samples was calculated using PCoA plots in MEGAN and NMDS plots (Bray-Curtis index, *p* < 0.005) in PAST. The correlation between environmental parameters and archaeal taxa was tested using linear unconstrained PCA model by selecting default Canoco advisor settings in Canoco 5 (version 5.10) [35]. The difference between archaeal communities in contaminated and agricultural site was further validated using ANOSIM in PAST. The canonical correspondence analysis (CCA) of environmental variables and operational taxonomic units was performed using PAST with the Bray-Curtis similarity index.

### 2.5. Archaeal Communities Predictive Metabolic Profiling

The predictive functional profiles of archaeal communities were inferred by using PICRUSt version 1.4.1 [36]. To enhance the accuracy of predictive profiles specific to archaeal communities, high-throughput sequencing reads of classified archaeal OTUs (from RDPclassifier) were extracted manually. QIIME [37] module in galaxy server [38] was used for closed-reference out picking to fulfill the PICRUSt pipeline criteria followed by copy number normalization, metagenome prediction, and functional characterization using KEGG pathway at different hierarchy levels. Data from the PICRUSt in the biom format was exported to STAMP [39] for graphical representation.

## 3. Results

### 3.1. Physicochemical Analysis of Soil

From SITE1 (*n* = 5 samples) and SITE2 (*n* = 9 samples), a total of 14 samples were subjected to pH, nitrate, electrical conductivity (EC), RDX, and HMX concentration estimation. Briefly, the concentration of RDX and HMX in SITE1 samples ranges from 56–165 mg/kg and 1–13 mg/kg, respectively, while the concentration of RDX and HMX in SITE2 soil was 0.2–157 mg/kg and 0.2–175 mg/kg, respectively. The soil samples from SITE1 and SITE2 (PS1, PS2, PS3, PS5, and PS7) showed high levels of RDX/HMX (>24 mg/kg) contamination. The pH, nitrate and E.C. were in the range of 2.5–5 and 3.5–7.1, 2.4–6.9 and 0.8–6.4 (mg/l), and 0.10–0.30 and 0.10–0.45 (mS/cm) in SITE1 and SITE2 samples, respectively. The pH levels in C1 and C2 were in the range of 6.2–6.3.

### 3.2. Diversity of Archaeal Community across SITE1 and SITE2

The archaeal population comprised a broad diversity of taxa across SITE1 and SITE2 samples. The alpha diversity (Shannon index) was greater on average in soil samples collected from SITE1 than SITE2 (Figure S1; Table S1). By contrast, the alpha diversity (Shannon index) was higher in control samples than SITE1 and SITE2 (Figure S1). In SITE1 maximum species diversity (Chao-1) was observed in NS3, while PS7 showed greater diversity in SITE2 samples (Table S1). The evenness of archaeal communities was considerably dissimilar in both sites, and SITE2 samples showed, on average, less evenness in archaeal population (Table S1). The ratio of total relative abundance of *Archaea* varied in average between the two sites and among the samples (Figure S2A,B). The total relative *Archaea: Bacteria* ratio in SITE2 (2.3:97.7) soil samples was lower than SITE1 (3.8:96.2) and control samples (2.9:97.05) (Figure S2A). Among SITE1 and SITE2, samples NS2 and PS9 showed highest relative abundance of *Archaea* (Figure S2B). The *Bacteria: Archaea* ratio was relatively higher in samples with higher RDX contamination (NS2, NS3), while in SITE2, samples with low HMX concentration (PS9, PS6) had high *Bacteria: Archaea* ratio (Figure S2B).

### 3.3. Archaeal Community Structure

**Figure 1:**
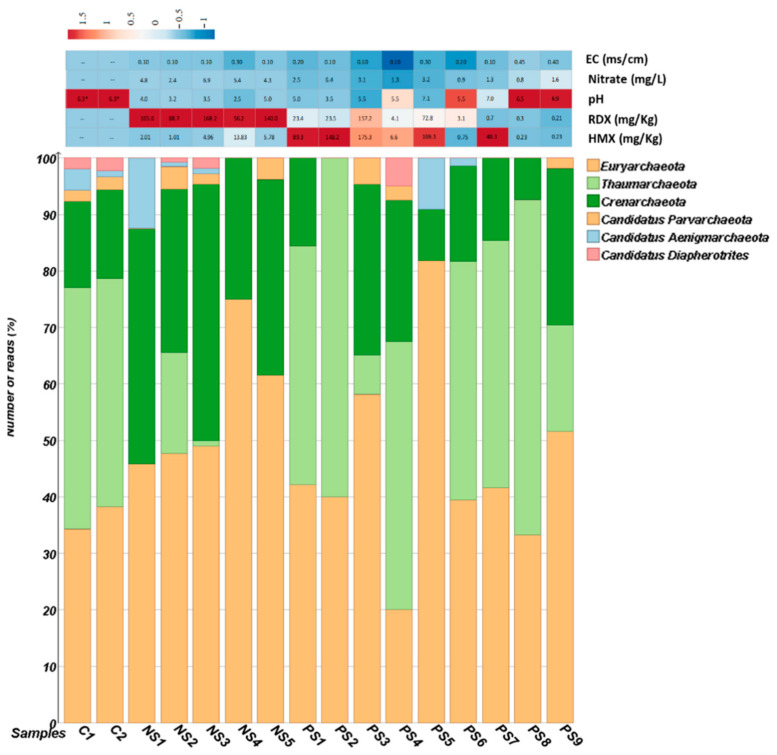
Dual bar chart represent phylum level distribution with levels of HMX, RDX and nitrate in samples collected from both sites. (*) represents data obtained from [26]. (--) represents data not available.

For a total of 14 samples in SITE1 and SITE2, the numbers of processed reads were in the range of 71,420–1,015,934 per sample and the numbers of the total observed OTU’s were in the range of 247–6568 per sample (Table S2). The number of OTUs identified as *Archaea* were in the range of 7–208 per sample, and in total 981 OTUs were identified as archaeal taxa in both sites. In the control samples (C1 and C2) the number of processed reads were in the range of 866,059–962,822 per sample and the total observed OTUs classified as *Archaea* were 614. The archaeal communities were dominated by *Euryarchaeota* (~47%), *Crenarchaeota* (~27%), and *Thaumarchaeota* (~22%) followed by small proportions of (~4%) *Candidatus* Parvarchaeota, *Candidatus* Aenigmarchaeota*,* and *Candidatus* Diapherotrites in SITE1 and 2. Moreover, the relative abundance of these major archaeal groups varied among these two sites. The relative abundance of *Euryarchaeota* and *Crenarchaeota* together constituted ~91% of archaeal population in SITE1, while *Euryarchaeota* and *Thaumarchaeota* constitute ~81% of relative archaeal diversity in SITE2 samples (Figure 1). By contrast, in the control samples *Thaumarchaeota, Euryarchaeota* and *Crenarchaeota* together constitute ~91% of archaeal diversity (Figure 1).

The PCoA plots based on Weighted-Uniform-Unifrac (phylum level) showed two distinct groups between SITE1 and SITE2 samples, however, sample PS3 and PS9 group with SITE1 samples, and C1 and C2 with SITE2, and sample PS4 and PS5 were the outliers (Figure S3). At the class level *Thermoprotei* (~37%) was more abundant in SITE1 samples, while in SITE2 and control samples *Nitrososphaeria* (~36%) was more dominant (Figure S4A–C). Interestingly, class *Methanobacteria* was not observed in SITE1 samples and was observed at significant levels only in a single sample from SITE2 (PS8) (Figure S4A). The archaeal communities belonging to family *Halobacteriaceae* were most dominant in SITE1 and *Nitrososphaeraceae,* in SITE2 and control samples (Figure S5). The major distinction at the family level was observed in terms of lack of *Methanocellaceae*, *Methanobacteriaceae*, and *Nitrosopumilaceae* in SITE1 samples (Figure S5). Interestingly, in control samples *Nitrosopumilaceae* was found abundant (~6.7%). Meanwhile, the top three most abundant genera at both (SITE1 and 2) sites belonged to *Nitrososphaeria* (~21%), *Salarchaeum* (~9.8%), and *Halocalculus* (~6%) (Figure S6). These two sites were also unique in their core-biome (taxa that are present in each sample of a considered site) composition. In SITE2, 12 taxa were found to represent the core-biome composition among which *Nitrososphaeria,* and *Haloquadratum* were unique to SITE2, while in SITE1, 18 taxa were identified, among which 9 taxa (*Candidatus* Aenigmarchaeum, *Candidatus *Parvarchaeum, *Caldisphaera*, Caldivirga,* Methanimicrococcus, Methanosalsum, Salinirubrum, Stygiolobus*, and *Thermocladium*) were found unique (Figure S7). In control samples, 26 taxa were found to represent the core-biome composition among which 14 taxa (*Candidatus* Iainarchaeum*, Cuniculiplasma, Ferroglobus, Ferroplasma, Halanaeroarchaeum, Halomicrococcus, Hyperthermus, Methanosphaerula, Methermicoccus, Nitrosopumilus, Pyrolobus, Sulfodiicoccus, Thermogladius* and *Thermosphaera*) were unique to control samples (Figure S7).

### 3.4. Archaeal Networks

**Figure 2:**
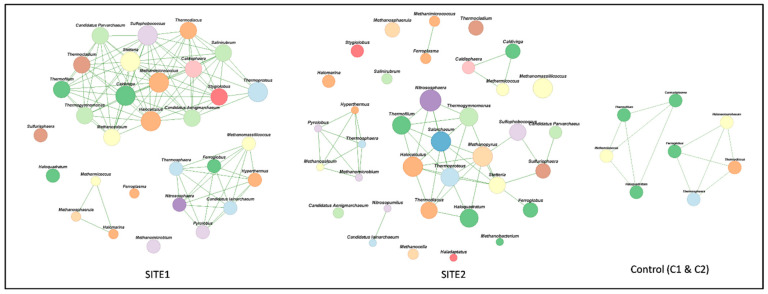
Co-occurrence networks from SITE1, SITE2 and agricultural soil samples. The networks were based on archaeal OTUs. Green lines indicate positive co-occurrence. Image was constructed in MEGAN [33] using Jaccard correlation coefficient with an edge threshold of 70%.

The co-occurrence network analysis showed that the association between the archaeal communities in SITE1 and SITE2 is distinct at the genus level. Greater significant relationships were observed in SITE1 samples (26 taxa with 126 correlations) compared to SITE2 samples (26 taxa with 48 correlations) (Figure 2). Further, associations between *Candidatus* Aenigmarchaeum (6 associations) and other taxa were only observed in SITE1 samples. Meanwhile, the key archaeal taxa in both sites with highest number of associations belonged to *Crenarchaeota* and *Euryarchaeota*. Interestingly, two distinct groups were observed for taxa representing phylum *Thaumarchaeota* (4 associations) in the SITE2 samples compared to a single group in SITE1 (Figure 2). By contrast, in the control samples only 8 taxa with 12 correlations were observed.

### 3.5. Comparison of the Archaeal Community in Explosives Contaminated and Agricultural Soil

**Figure 3:**
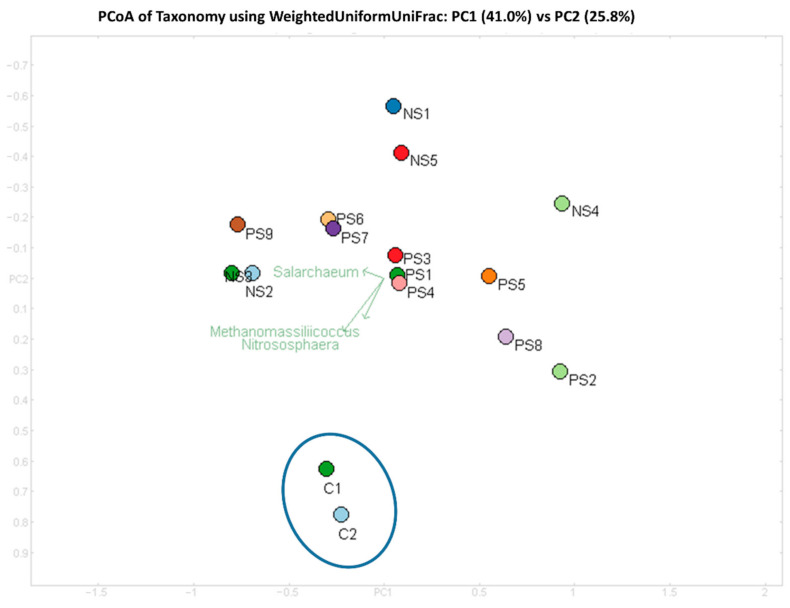
PCoA of samples at genus level using Weighted-Uniform-UniFrac distance. Triplot arrow represent the projection of genera for respective samples. Image was constructed in MEGAN [33].

Samples from the present study were also compared with agricultural soil samples (designated as C1 and C2) to differentiate the archaeal community structure in explosives contaminated soils versus agricultural soil samples. The archaeal community in contaminated soil samples was clearly different from agricultural soil samples and clustered separately from SITE1 SITE2 samples (Figure 3).

**Figure 4:**
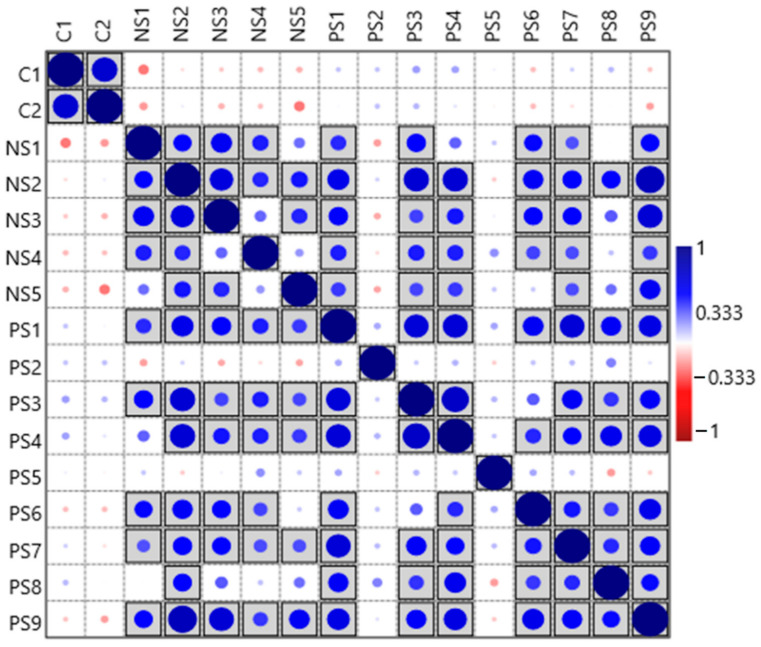
Correlation plot between samples from explosives contaminated (SITE1 and SITE2) and agricultural soil (C1, C2) at genus level. Correlation plot was based on Spearman rank correlation. Grey highlighted squares represent significance, *p* ≤ 0.005. Filled circles (size) represent correlation between the samples. Image was constructed in PAST software [30].

Moreover, the findings of ANOSIM statistics further validated a clear distinction between archaeal communities in explosives contaminated and agricultural soil samples (R = 0.8592, *p* = 0.0001). Further, the correlation plots (Spearman rank correlation, *p* ≤ 0.005) showed no significant relation between samples from two environments (Figure 4) and suggest that archaeal composition is different in contaminated and agricultural site.

**Figure 5:**
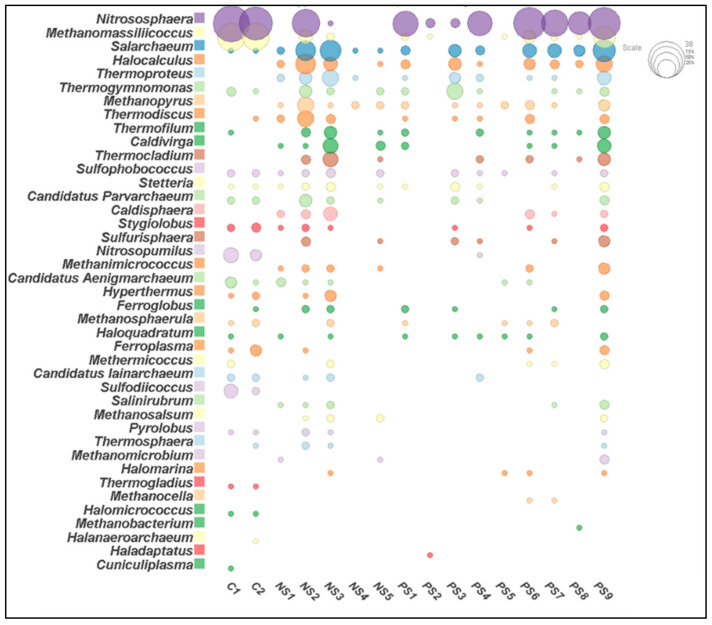
Bubble plot representing genus level distribution in control (C1, C2) and explosives contaminated (NS1-PS9) samples. The scale represents the percentage at the genus level. Image was constructed in MEGAN [29] software.

When the archaeal communities were compared at genus level between the contaminated and agricultural soil samples striking differences were observed. A total of 15 genera (*Caldisphaera, Caldivirga, Haladaptatus, Halocalculus, Halomarina, Methanimicrococcus, Methanobacterium, Methanocella, Methanomicrobium, Methanopyrus, Methanosalsum, Salinirubrum, Sulfurisphaera, Thermocladium,* and *Thermoproteus*) were unique to SITE1, and SITE2 samples, while *Cuniculiplasma, Halanaeroarchaeum, Halomicrococcus, Thermogladius, Sulfodiicoccus* were unique to agricultural soil samples (Figure 5).

### 3.6. Predictive Archaeal Metabolic Profiles

The predictive metabolic profiles of archaeal communities resulted in 203 functional pathways. Metabolism of *amino acid*, *carbohydrate*, *energy*, *translation*, and *membrane transport* were the top five metabolic pathways in archaeal communities observed in SITE1 and SITE2. In addition, the pathways involved in the *membrane transport*, *energy* and *lipid metabolism* were more dominant in agricultural soil samples. Pathways responsible for the metabolism of *xenobiotic biodegradation* were identified in both sites (SITE 1 and SITE 2; Figure S8) and in higher proportions compared to the agricultural soil samples. However, the pathways for metabolism of xenobiotics by *cytochrome* P450, *xylene* and *atrazine* degradation were in greater abundance in SITE2 samples (data not shown).

## 4. Discussion

Physicochemical parameters and microbial factors [40,41] often govern the degradation of explosives in soil and groundwater. Several culture independent studies have revealed the bacterial community structures at various explosives contaminated sites [23,42,43,44,45], though knowledge on the role and association of archaeal communities is still rudimentary. The present work gives a broader view of archaeal community composition and their metabolic profiles at two geographically distinct explosives contaminated sites. To our knowledge, this is the first *Archaea*-specific metabarcoding analysis of explosive contaminated environments.

The degree of explosives contamination and other prevalent factors were highlighted by physicochemical analysis of samples. The soil samples from SITE1 and SITE2 (PS1, PS2, PS3, PS5, and PS7) showed high levels of RDX/HMX (> 24 mg/kg) contamination, which surpasses both the U.S, EPA residential soil screening levels (SSL) (5.6 mg/kg) and industrial screening level (ISL, 24 mg/kg) [46] limits. These elevated levels highlight the extent of contamination at the sites and the potential of RDX and HMX to migrate in soil by virtue of low sorption capacity [47]. Further, the nitrate-nitrogen (32–96 mg/kg) and ammoniacal nitrogen (240–875 mg/kg) were detected in high quantities in our samples that could be a result of RDX and HMX degradation by photolysis/alkaline hydrolysis or microbiological activities resulting in the release of nitrate/nitrite/ammonia in the environment [48,49,50]. Overall, the physicochemical parameters indicated extreme conditions with respect to pH and xenobiotic compounds, suitable for the success of archaeal communities [51,52].

The disparity in *Bacteria: Archaea* ratios and differences between SITE1 and SITE2 samples is noticeable (Figure S2A). It was interesting to note that the prokaryotic communities varied considerably within the samples (Figure S2B). Surprisingly in SITE1 samples, higher *Bacteria: Archaea* ratio was observed in samples with higher RDX contamination (NS2, NS3). However, in SITE2, high *Bacteria: Archaea* ratio was observed in samples with low HMX concentration (PS9, PS6) (Figure S2B). A possible explanation could be a relatively complex structure of HMX and lower rates of photolysis compared to RDX, thus making it recalcitrant towards utilization by the microbial population [51,53]. In addition, pH and physiologically important elements like NH_4_, nitrate which lead to niche separation and differentiation, may possibly result in the diverse distribution of archaeal taxa in these sites [54,55,56].

The *Archaea* form a significant part of the ecological niche and account for >3.36% of the *Prokaryotes* on Earth [1,57] (https://www.arb-silva.de/documentation/release-138/)(accessed on 2 January 2022) thus, our study focused on the prevalence of these taxa. High-throughput sequencing analysis in our study suggested that the dominant OTU’s belonged to phyla *Euryarchaeota*, *Crenarchaeota,* and *Thaumarchaeota* (Figure 1) in SITE1, SITE2 and control samples. Surprisingly, a clear distinction was observed at the phylum level in samples contaminated with high concentrations of RDX (≥56–166 mg/kg), i.e., SITE1 and HMX (≥6–174 mg/kg), i.e., SITE2 (Figure 1) with a possible positive correlation between concentration of RDX and abundance of *Candidatus* Aenigmarchaeota (Figure S9). In addition, the environmental parameters RDX and nitrate were found more linked to SITE1 samples than pH and E.C which were observed more associated to SITE2 samples (Figure S9). Though the role of *Candidatus* Aenigmarchaeota is not very well understood in explosives contaminated sites, previous reports on their symbiotic relations have suggest their ability to thrive in environmental stresses, such as oxidative stress and high temperature [58].

**Figure 6:**
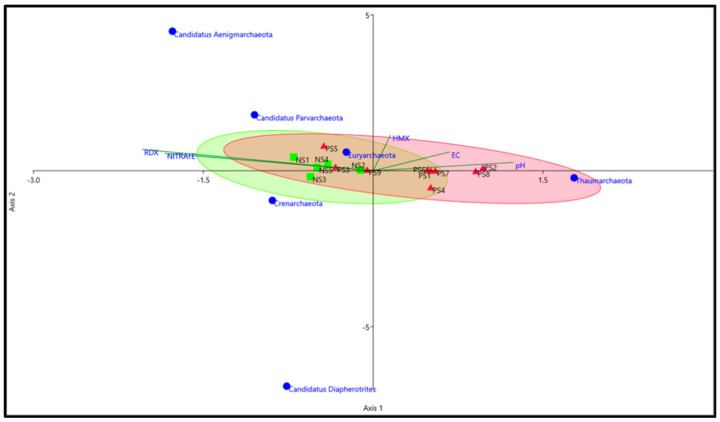
Canonical correspondence analysis (CCA) plot between environmental variables and phylum level diversity. Triplot represent environmental variables. Filled squares and triangles represent samples from SITE1 and 2, respectively. CCA plot was constructed based on bootstrap N:999, *p* = 0.043.

The correlation between concentration of RDX and abundance of *Candidatus* Aenigmarchaeota is also well supported by the CCA and NMDS plots of physicochemical parameters highlighting the abundance of *Candidatus* Aenigmarchaeota in samples with higher concentration of RDX and nitrate (SITE1), while members of *Thaumarchaeota* were more prevalent in samples with higher levels of pH and EC (SITE2) (Figure 6, Figures S9 and S10).

The predominance of *Euryarchaeota* in SITE1 and SIET2 samples was not surprising considering the fact that it forms about ~25% of the total archaeal population in the environmental samples [59], and members of *Euryarchaeota* (*Methanococcus* sp.) are known degraders of explosives [60]. In contrast, members of *Thaumarchaeota* are known for their potential to oxidize ammonia [61,62,63] and play a significant role in global nitrogen cycling [64]. Both nitrate and ammonia are important intermediates during microbial degradation of explosives [65], therefore, members of *Thaumarchaeota* can be of key importance in these habitats. Studies related to the role of archaeal communities in biodegradation showed more prominent abundance of *Crenarchaeota* in soil samples contaminated with crude oil [9] supporting a possible role of these lineages in the biodegradation of explosives.

The two most dominant classes in our samples, *Thermoprotei* and *Nitrososphaeria* (Figure S4), have not yet been reported from explosives contaminated sites. However, the available literature suggest *Thermoprotei* presence and role in hydrocarbon (crude oil) biotransformation while members of *Nitrososphaeria* (ammonia-oxidizing archaea) were reported to transform pharmaceutical products and to have a specific role in ammonia oxidation [66,67]. It is pertinent to mention here that ammoniacal nitrogen, a biodegradation product of explosives [64] was detected in high quantities in both SITE1 (875 mg/kg) and SITE2 (239 mg/kg) samples NS2 and PS2, respectively, contaminated with highest concentrations of explosives and this can possibly be linked to the abundant ammonia-oxidizing *Nitrososphaeria* (ammonia-oxidizing archaea) populations involved in nitrification in both sites [66,67]. Though the ammoniacal nitrogen data is not available for the control samples, it is also important to note that *Nitrososphaeria* are also found abundant in agricultural samples and further investigation with respect to physicochemical parameters will be important to highlight their role in agricultural soil. Our study also indicates the predominance of genera *Salarchaeum* and *Halocalculus* (both genera comprising only a single representative species) in SITE1 and SITE2 samples (Figure S6), which have not yet been isolated from contaminated sites and were previously identified only from commercial salt brines [68,69]. The prevalence of halophiles and methanogens have previously been associated with degradation of aliphatic-aromatic hydrocarbons and nitrate-driven oxidation of methane [70,71]. The available literature to date insinuates that role of these taxa in explosive contaminated habitats is still unrecognized.

The co-occurrence network analysis suggested that archaeal communities formed a complex network (26 taxa with 166 and 48 correlations respectively at SITE1 and SITE2), and the key taxa identified were mainly from *Desulfurococcaceae, Halobacteriaceae, Thermoproteaceae, Methanosarcinaceae, Pyrodictiaceae,* and *Methermicoccaceae*, while the major distinctiveness in SITE1 and SITE2 co-occurrence pattern was *Nitrosopumilus* and was only observed in SITE2 samples (Figure 2). Subsequently, a similar pattern was observed in the core biome profile where *Nitrososphaeria* was the major taxa in SITE2 and *Salarchaeum* in SITE1 (Figure S7). In addition, *Halocalculus*, *Salarchaeum*, and *Haloquadratum* within the class *Halobacteria* were found co-occurring with the methanogenic archaeal groups (i.e., *Methanopyrus*) at SITE2 (Figure 2) that are involved in nitrogen fixation and nitrogen cycling [72] and have been earlier reported to biotransform and metabolize RDX in Ovine ruminal fluid [73]. The archaeal communities have a profound effect on their proximity via interaction with biotic and abiotic environmental components, mainly detoxification, capability for exchange of metabolites and capacity for structural adaptation and has been well researched [74,75,76]. We speculate that these co-occurring taxa and core biome populations may have an imperative role in shaping the overall microbial profile in these contaminated sites.

The archaeal diversity of explosives contaminated sites was also compared with two agricultural soils [26] where two samples with no history of exposure to explosives (agricultural soil) were used for comparison. The archaeal diversity was considerably different between the agricultural soil and contaminated soil samples (Figure 5). The ANOSIM statistical analysis (R = 0.8592, *p* = 0.0001) suggests strong, statistically significant difference in the microbial communities of agricultural soil and contaminated samples. Further, the correlation plots (Spearman rank correlation, *p* ≤ 0.005) also suggested no significant relation between samples from both environments (Figure 4). The agricultural soil and contaminated sites (SITE1 and SITE2) were also found unique at the genus level, where 15 genera were found exclusive in our samples (Figure 5). Although no direct study is available to link presence of these genera in explosives contaminated sites however, some of these taxa, i.e., *Halocalculus, Methanopyrus, Methanomicrococcus, Sulfurisphaera, Thermocladium,* and *Thermoproteus* have previously been associated with hydrocarbon, organics degradation, and have been isolated from acid mine drainage, hypersaline, and acidic environments [10,65,77,78,79,80], highlighting the adaptability of these archaeal groups to thrive in such niches. In addition, all these genera were only observed in SITE1 and SITE2 samples and were not observed in the control (C1and C2) samples (Figure S6).

The predictive metabolic pathways of both the sites showed the abundance of *amino acid*, *carbohydrate*, *nucleotide metabolism*, *membrane transport*, *replication* and *repair*, *xenobiotics biodegradation*, metabolism of *terpenoids* and *polyketides* (Figure S8). Meanwhile, pathways for metabolism of xenobiotics by *cytochrome* P450, *xylene*, and *atrazine* degradation were in greater abundance in SITE2 samples. Expectedly, the pathways for *xenobiotic biodegradation* and metabolism could not be discerned in the control samples (Figure S8). Though at this point the archaeal community responsible for xenobiotic degradation pathways could not be directly discerned using predictive metabolic profiling, the available data indeed indicate potentially active degradation processes in explosives contaminated sites. The data from previous studies also indicate that microbial communities can sustain the environmental stress incurred by extreme conditions, to which the *Archaea* are evolutionarily adapted [81]. Future efforts could be directed to assess the targeted archaeal communities using meta-transcriptomics, culturable, and whole metagenome approaches and how these communities specifically respond to environmental stress.

## 5. Conclusions

For the present study, we analyzed and compared the archaeal distribution, diversity, network, and potential metabolic pathways in soil samples from two distinct explosives contaminated sites using Archaea-specific, 16S amplicon high-throughput sequencing. Our results suggest distinct distribution of archaeal communities at the explosives contaminated sites and indicate a substantial influence of abiotic factors on archaeal abundance. In addition, significant disparity in the pattern of archaeal communities in explosives contaminated and agricultural sites was also observed. These archaeal communities have numerous metabolic pathways and complex co-occurring networks which perhaps aids in proliferation of archaeal communities in contaminated sites and their potential degradative metabolism. Collectively, our research suggests that explosives contaminated sites have a unique archaeal community profile that require further introspection through conventional culturomics and other omics based approaches to understand their role in explosive degradation directly and through interaction with bacterial communities.

## Data Availability

The raw sequencing reads data is available at NCBI SRA database under Bioproject PRJNA391401.

## Supplementary Materials

The following supporting information can be downloaded at: https://www.mdpi.com/article/10.3390/biom12040489/s1, Figure S1: The image represent collective Shannon diversity in SITE1, SITE2 and control samples; Figure S2: (A) OTU based relative Bacteria and Archaea ratio in SITE1, SITE2 and control samples; (B) OTU based relative Bacteria and Archaea ratio between SITE1, SITE2 and control samples; Figure S3: PCoA plot of samples at phylum level using Weighted-Uniform-UniFrac distance; Figure S4: Bar chart represent class level distribution. (A) Represent SITE1; (B) SITE2; and (C) Control samples; Figure S5: Bar chart represent family level distribution. (A,B) Represent SITE1; SITE2; and (C) Control samples; Figure S6: Image represent genus level distribution in Control, SITE1 and SITE2 samples; Figure S7: Bubble plot represent core-biome composition in SITE1, SITE2 and control samples; Figure S8: Heatmap represent distribution of metabolic pathways in SITE1, SITE2, and Control (uncontaminated samples C1 & C2) based on KEGG pathways (Hierarchy level 2); Figure S9: Unconstrained PCA plot constructed using linear ordination method represents environmental variables and the archaeal taxa; Figure S10: Non-metric multidimensional scaling (NMDS) plot at phylum level; Table S1: Table represent different diversity Indices for SITE1, SITE2 and control samples; Table S2: Table represents the total number of paired end reads, processed reads, total number of OTU’s picked and the identified archaeal OTUs in SITE 1, SITE 2 and control samples.
