# Supplementary material for: Exploring the Distinct Distribution of Archaeal Communities in Sites Contaminated with Explosives
Source: Biomolecules. 2022 Mar 23;12(4):489. doi: 10.3390/biom12040489 (PMC9028785; doi:10.3390/biom12040489)
Supplement: Supplementary file 1 [file biomolecules-12-00489-s001.zip › biomolecules-1429069-supplementary.pdf]

Table S1. Table represent different diversity Indices for SITE1, SITE2 and control samples.

S. No	Sample ID	Simpson_1-D	Shannon_H	Evenness_e^H/S	Chao-1
	SITE 1				
1	NS1	0.9654	2.308	0.6703	21
2	NS2	0.9256	2.747	0.5775	29.63
3	NS3	0.9502	2.919	0.6387	31.55
4	NS4	0.8333	0.7897	0.7343	3.5
5	NS5	0.9632	2.229	0.6634	21.2
	SITE 2				
6	PS1	0.7875	1.896	0.4756	15.43
7	PS2	0.7	0.7503	0.7059	4
8	PS3	0.915	2.24	0.6708	16.5
9	PS4	0.7841	1.911	0.4223	25
10	PS5	0.9643	1.531	0.6605	14.5
11	PS6	0.7967	2.081	0.4008	38.33
12	PS7	0.7872	1.951	0.3909	44
13	PS8	0.6382	1.266	0.4435	13
14	PS9	0.9251	2.848	0.5948	29.43
	CONTROL				
15	C1	0.7968	2.169	0.3978	31.17
16	C2	0.7999	2.223	0.4017	32.43

Table S2. Table represents the total number of paired end reads, processed reads, total number of OTU's picked and the identified archaeal OTUs in SITE 1, SITE 2 and control samples.

Sample Name	Total paired end reads	Processed reads	Total OTU's observed	Identified Archaeal OTU's
**Amplicon data of 16S rRNA (V3-V4) (with Archaea specific primers)**				
N.S1	863002	597959	825	30
N.S2	1321670	1015934	6568	175
N.S3	879177	660790	2428	166
N.S4	96762	71420	247	7
N.S5	549749	419619	709	32
P.S1	316699	240027	2796	59
P.S2	432244	271311	2834	8
P.S3	740513	512931	1638	63
P.S4	356945	272748	3359	56
P.S5	242797	171727	354	18
P.S6	358931	265078	3189	78
P.S7	328031	255932	2926	53
P.S8	257147	193803	1585	28
P.S9	878478	618450	6300	208
C1	1084424	962822	10750	332
C2	985392	866059	9939	282

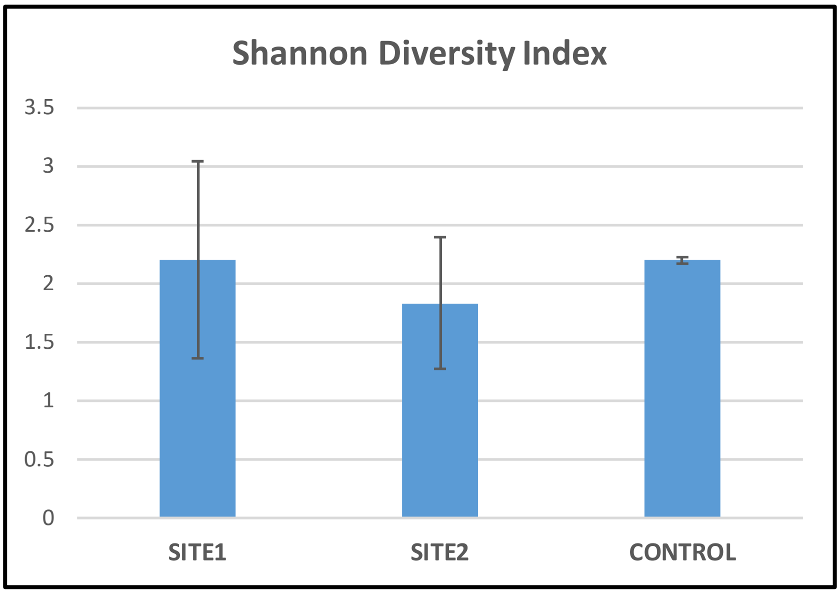

Figure S1. The image represents collective Shannon diversity in SITE1, SITE2 and control samples. Error bars represent standard deviation.

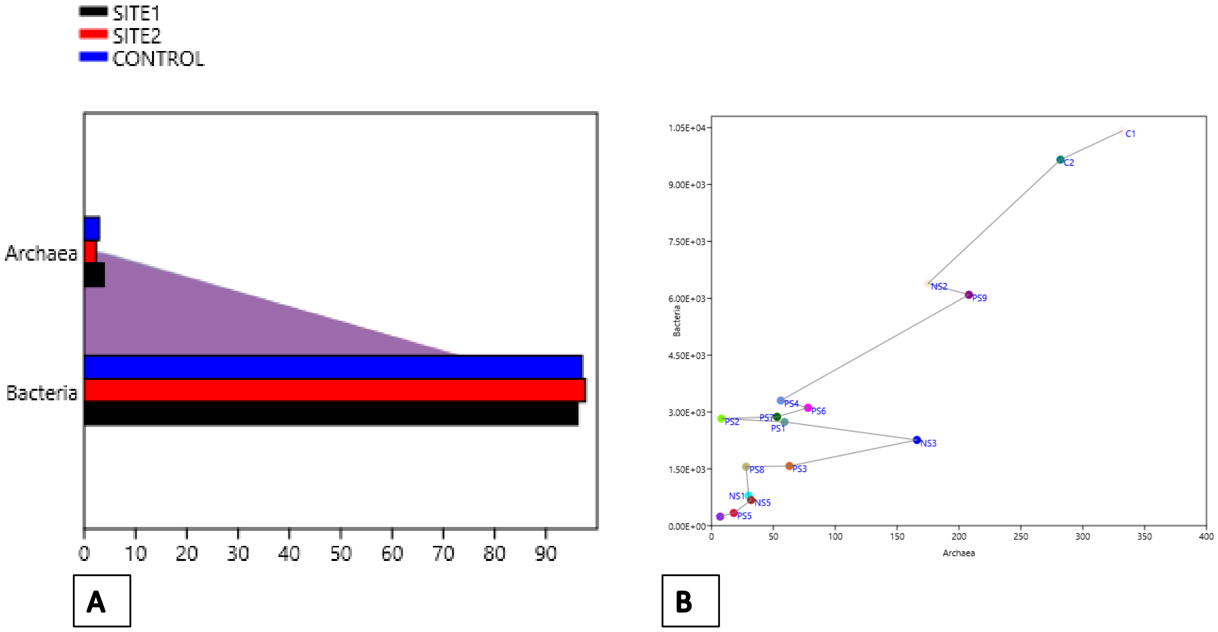

Figure S2. (**A**) OTU based relative *Bacteria* and *Archaea* ratio in SITE1, SITE2 and control samples; (**B**) OTU based relative *Bacteria* and *Archaea* ratio between SITE1, SITE2 and control samples. Image was constructed using PAST software.

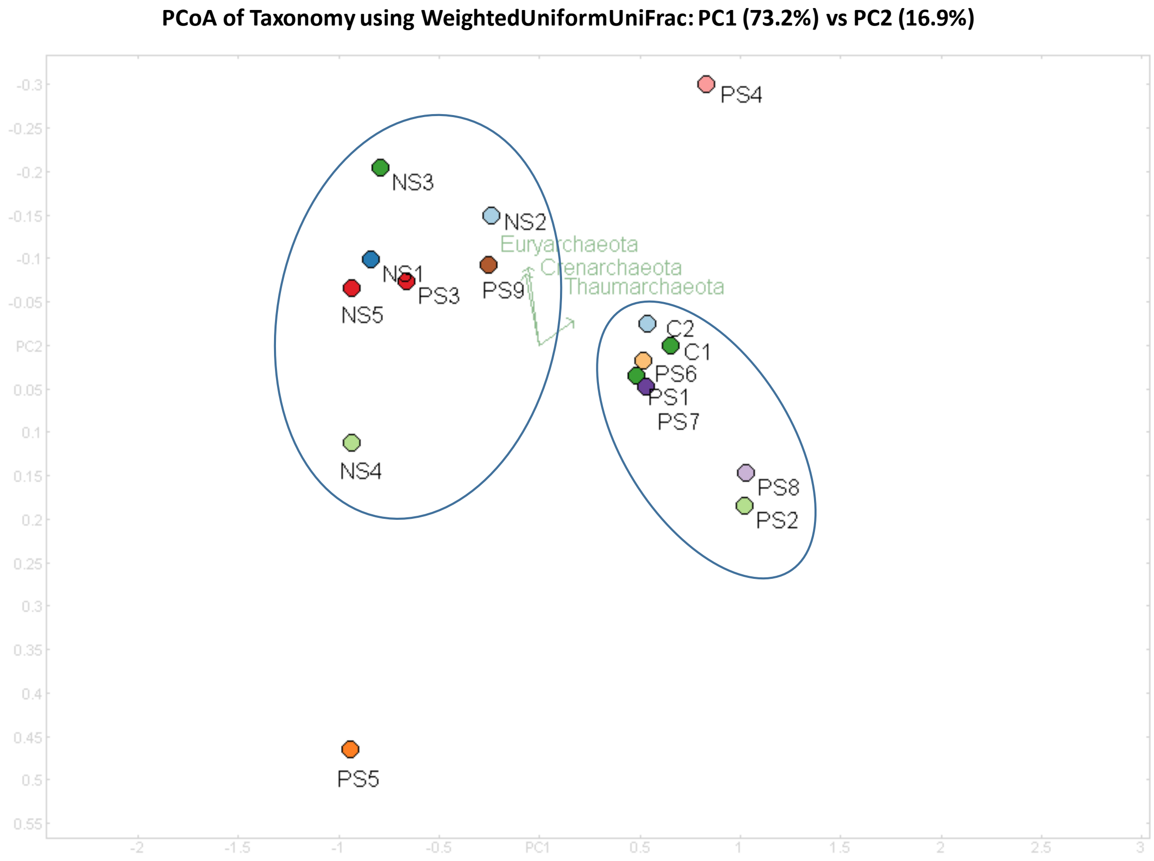

Figure S3. PCoA plot of samples at phylum level using Weighted-Uniform-UniFrac distance. Triplot arrow represent the projection of phylum for respective samples. Images were constructed in MEGAN.

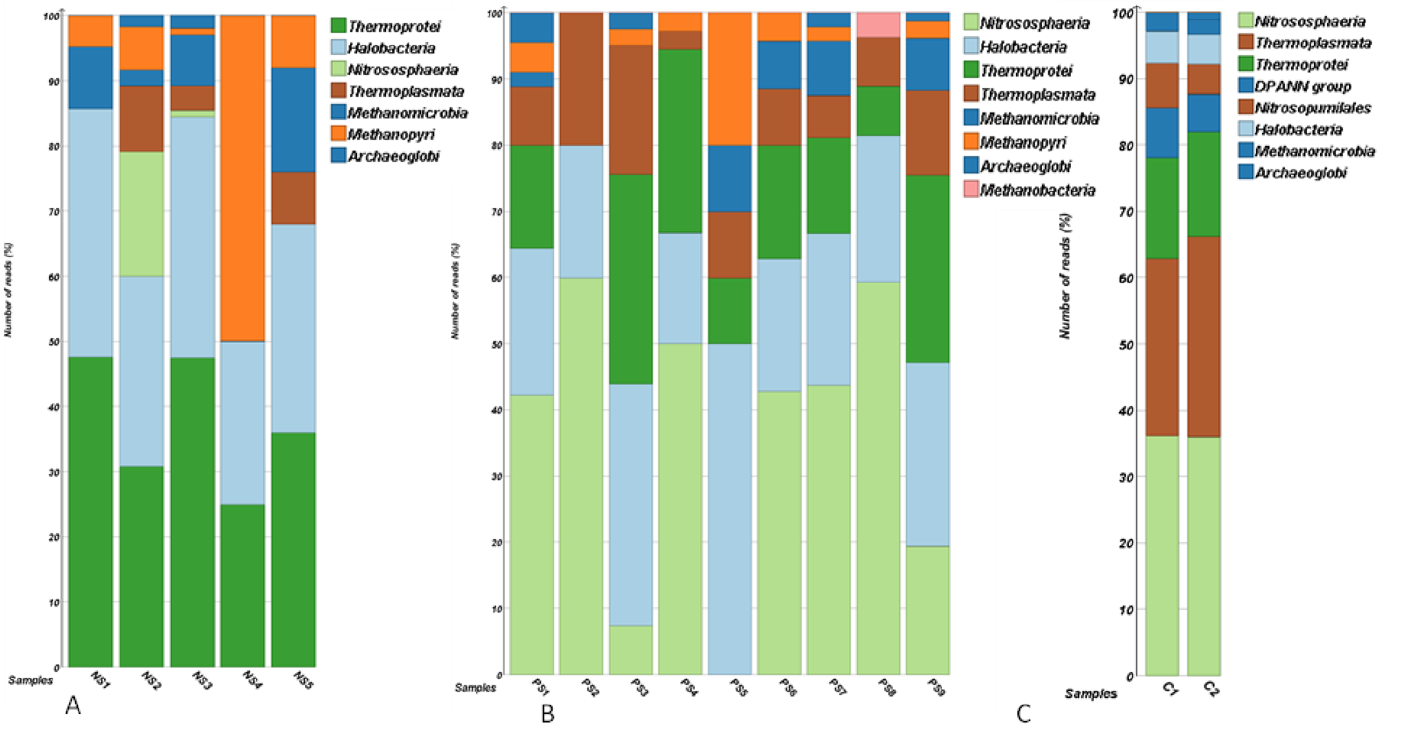

Figure S4. Bar chart represent class level distribution. A) Represent SITE1; B) SITE2; and C) Control samples.

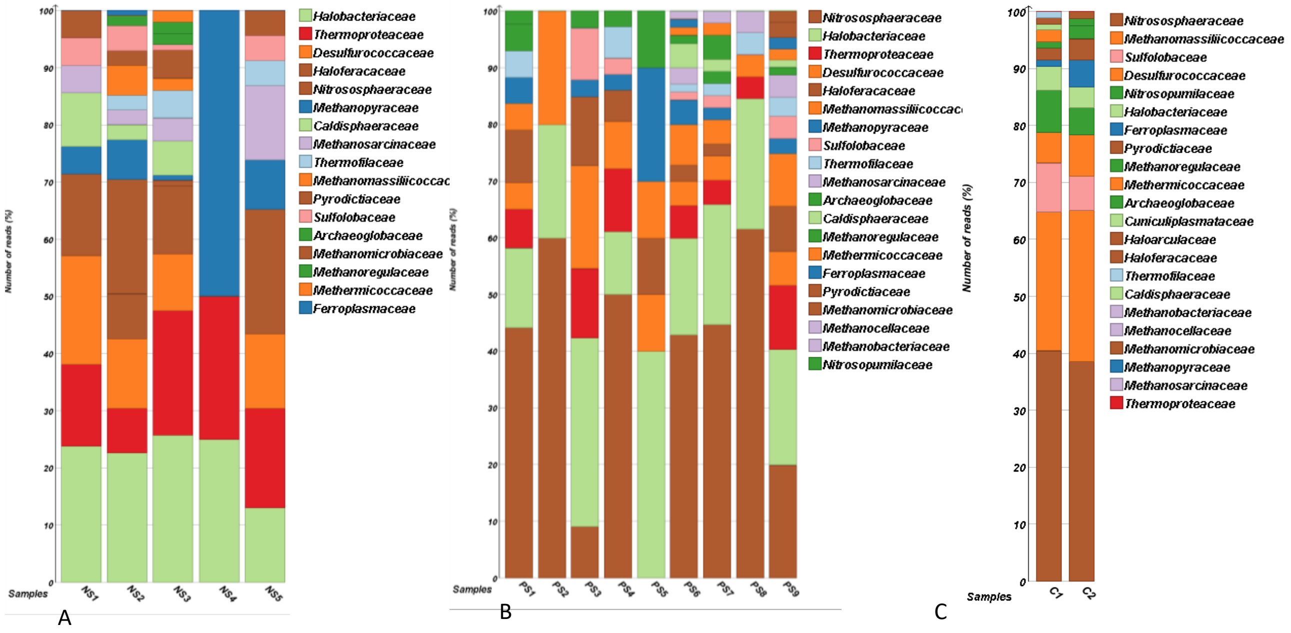

Figure S5. Bar charts represent family level distribution. (**A**,**B**) Represent SITE1; SITE2; and (**C**) Control samples.

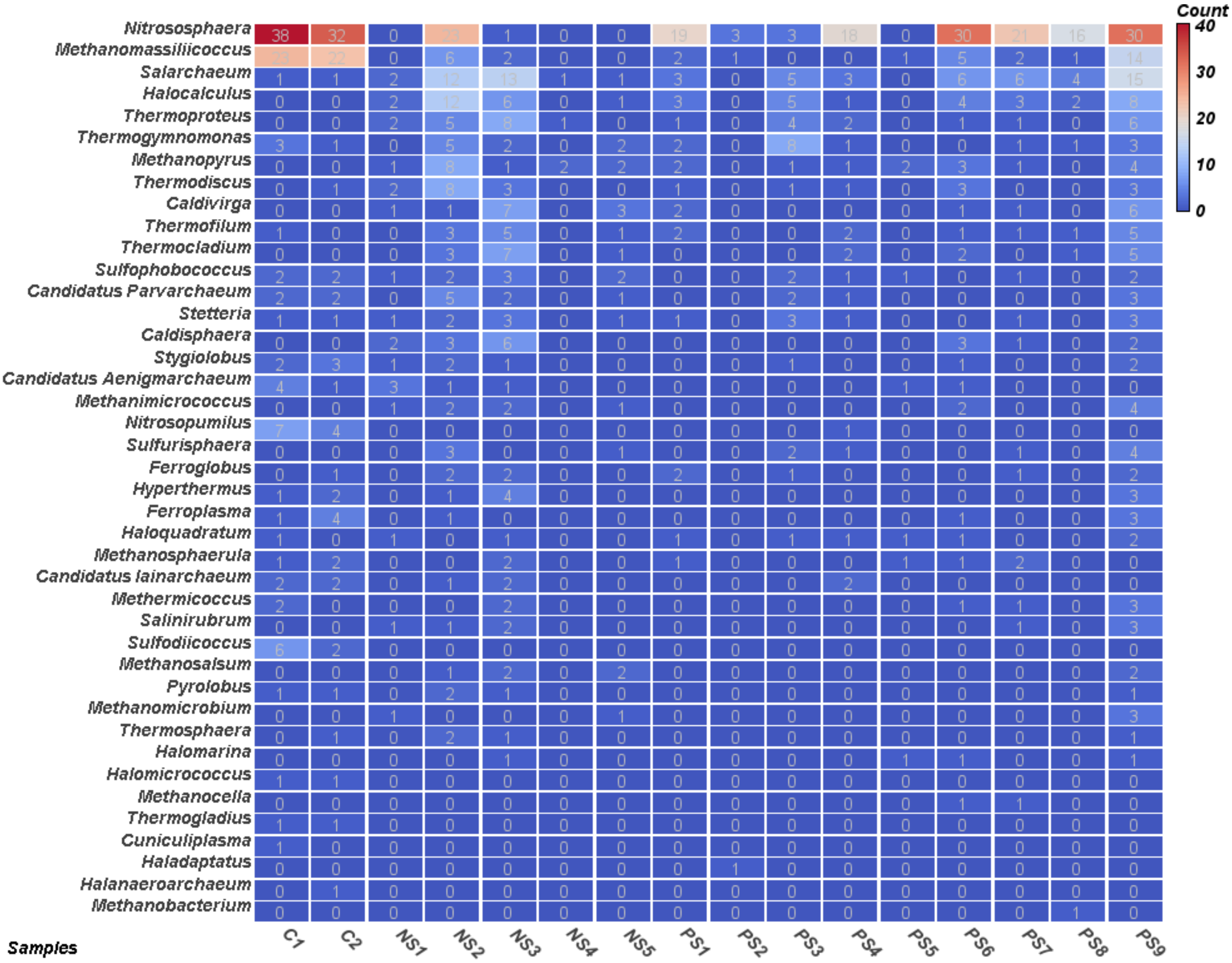

Figure S6. Image represents genus level distribution in Control, SITE1 and SITE2 samples. Legends represent the respective OTU counts.

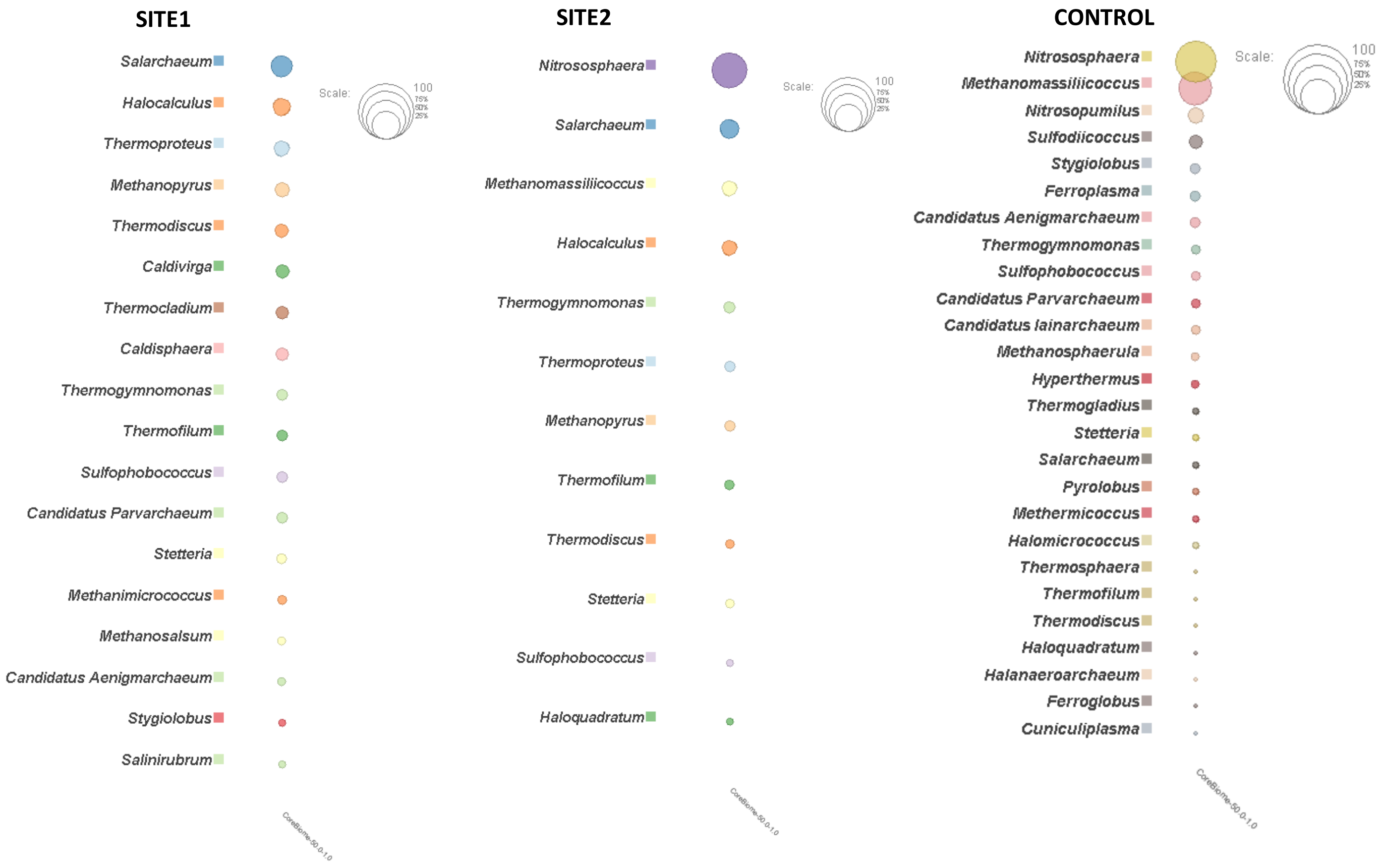

Figure S7. Bubble plot represents core-biome composition in SITE1, SITE2 and control samples. Scale represents percent distribution for respective taxa.

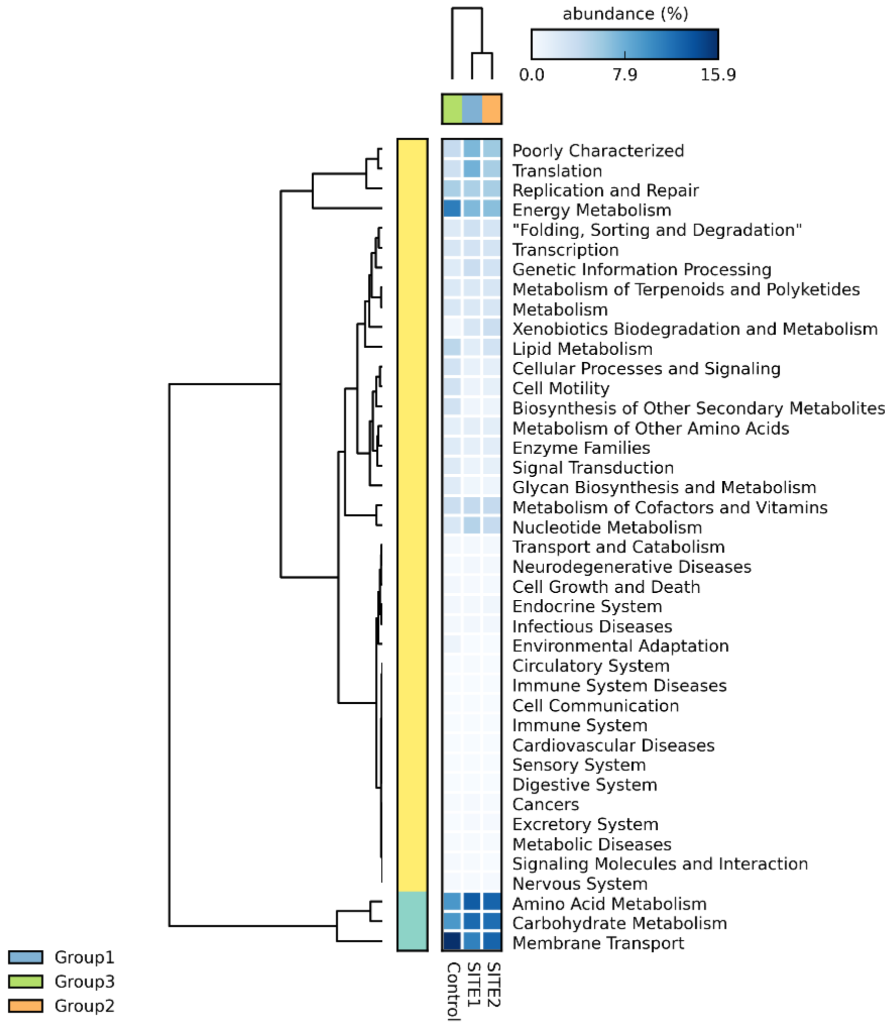

Figure S8. Heatmap represent distribution of metabolic pathways in SITE1, SITE2, and Control (uncontaminated samples C1 and C2) based on KEGG pathways (Hierarchy level 2). Image was constructed based on data from PICRUSt pipeline in STAMP software.

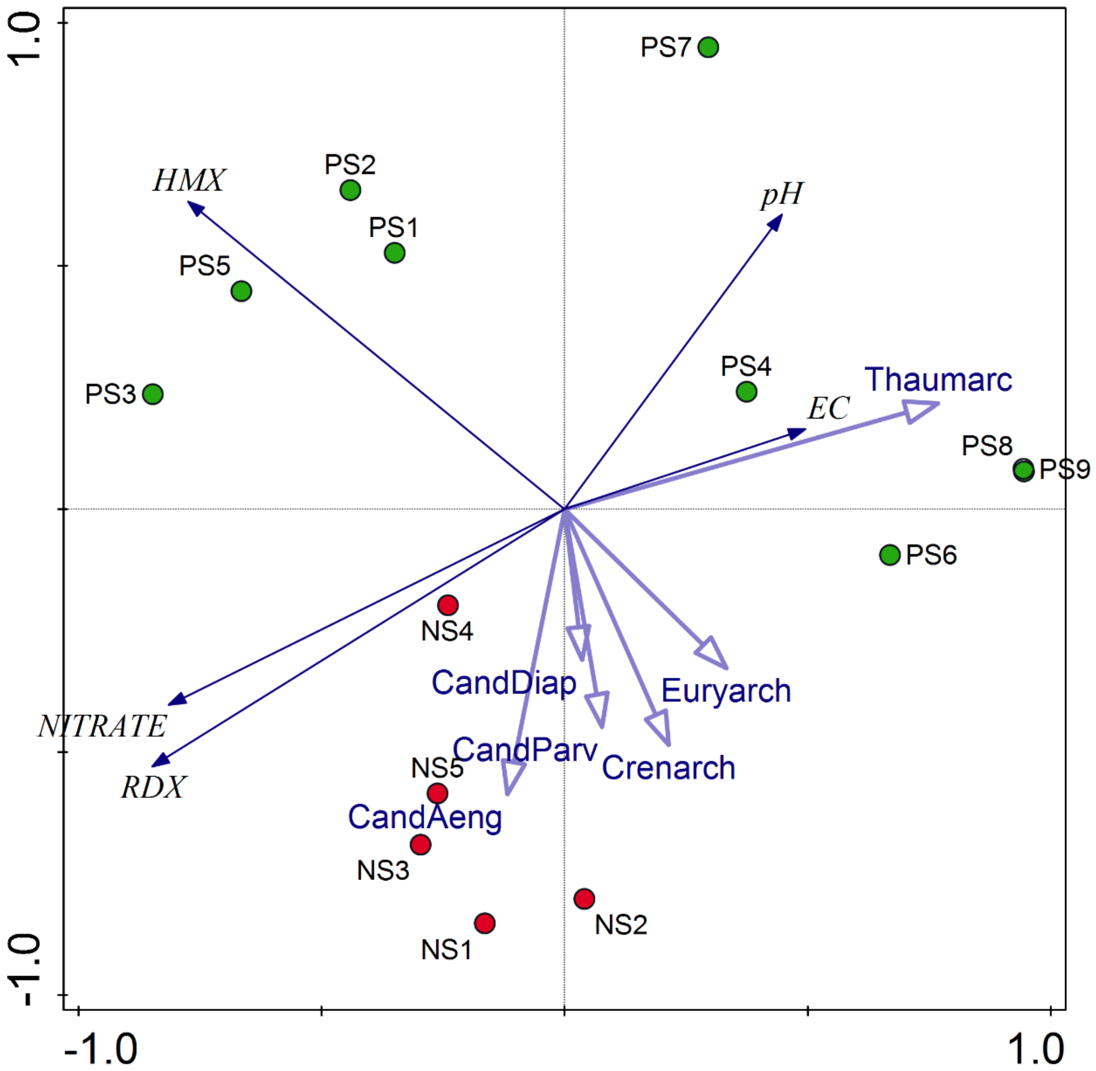

Figure S9. Unconstrained PCA plot constructed using linear ordination method represents environmental variables and the archaeal taxa. Filled dots represent samples from SITE1 and SITE2. Image was constructed in Canoco version 5.10 [35].

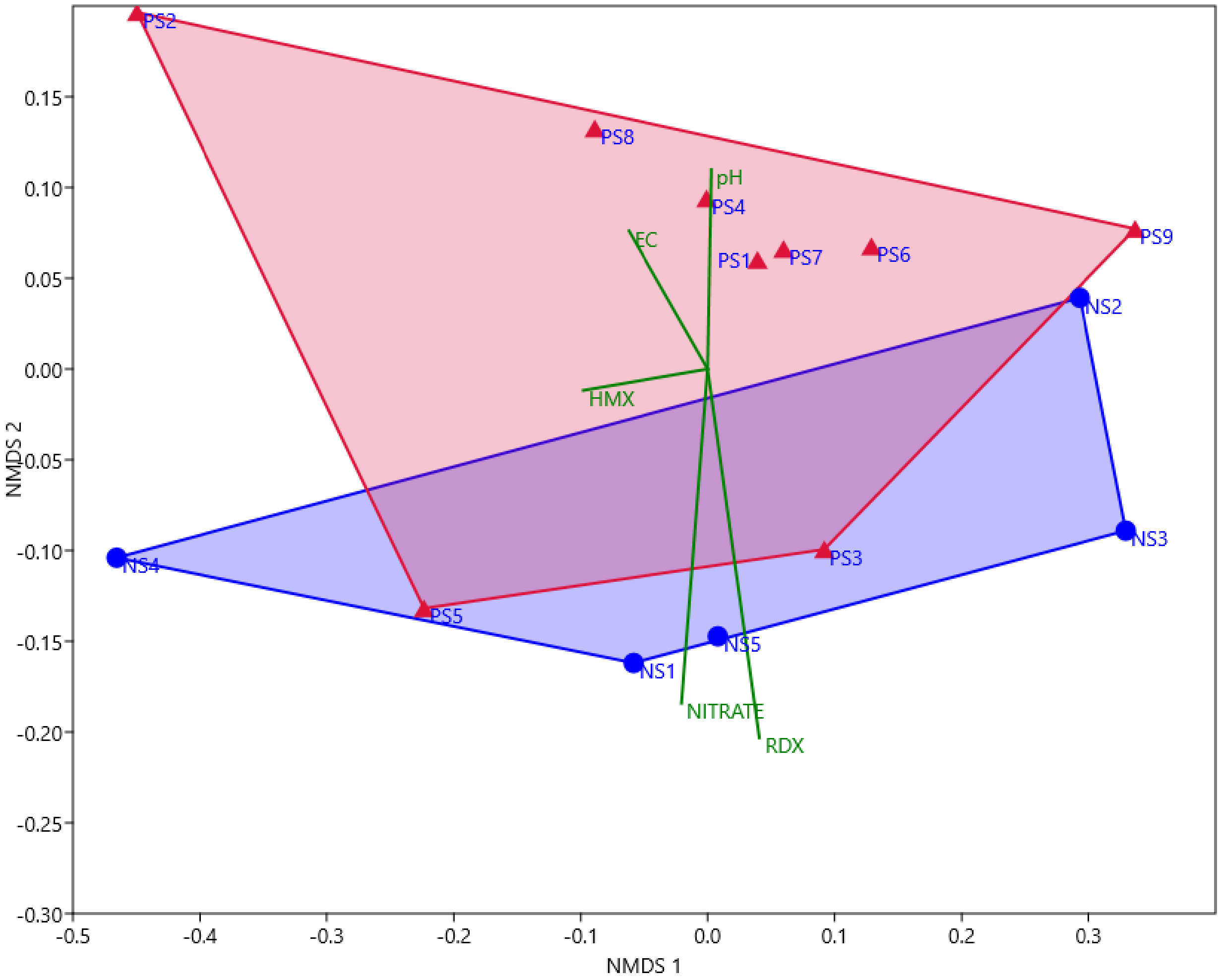

Figure S10. Non-metric multidimensional scaling (NMDS) plot at phylum level. Triplot represent environmental variables. Filled triangles represent samples from SITE2 filled squares represent samples from SITE1. NMDS was based on Bray-Curtis index.
